# Determinants of and Barriers to Hormonal and Surgical Treatment Receipt Among Transgender People

**DOI:** 10.1089/trgh.2016.0013

**Published:** 2016-07-01

**Authors:** R. Craig Sineath, Cory Woodyatt, Travis Sanchez, Shawn Giammattei, Theresa Gillespie, Enid Hunkeler, Ashli Owen-Smith, Virginia P. Quinn, Douglas Roblin, Robert Stephenson, Patrick S. Sullivan, Vin Tangpricha, Michael Goodman

**Affiliations:** ^1^ Rollins School of Public Health, Emory University, Atlanta, Georgia.; ^2^ The Rockway Institute, Alliant International University, San Francisco, California.; ^3^ School of Medicine, Emory University, Atlanta, Georgia.; ^4^ Atlanta VA Medical Center, Decatur, Georgia.; ^5^ Division of Research, Kaiser Permanente, Oakland, California.; ^6^ School of Public Health, Georgia State University, Atlanta, Georgia.; ^7^ School of Nursing University of Michigan, Detroit, Michigan.; ^8^ Kaiser Permanente Southern California, Los Angeles, California.

**Keywords:** barriers to care, epidemiology, transgender

## Abstract

**Purpose:** Medical gender confirmation therapy (GCT) plays an important role in transgender health; however, its prevalence and determinants constitute an area of uncertainty.

**Methods:** Data for this cross-sectional study were obtained from an online survey distributed from October 2012 through the end of 2013 among persons who visited the social media sites of a transgender education and social networking meeting. Eligible respondents (*n*=280) were persons whose gender identity was different from their sex assigned at birth and who responded to questions about previously received or planned hormonal therapy (HT), chest reconstruction, or genital surgery. Multivariable logistic regression models examined how receipt and plans to receive different GCT types were associated with participants' characteristics and gender identity.

**Results:** The respective percentages of ever and current HT were 58% and 47% for transwomen and 63% and 57% for transmen. Genital surgery was reported by 11 participants; all transwomen. Relative to transmen, transwomen were thrice more likely to report plans to undergo genital surgery. By contrast, transmen were more than 10 times as likely as transwomen to have had or planned chest surgery. Older participants and those who were in a committed relationship were less likely to plan future GCT. Having health insurance was not associated with GCT receipt. Treatment cost was named as the main problem by 23% of transwomen and 29% of transmen. Accessing a qualified healthcare provider for transgender-related care was listed as the primary reason for not receiving surgery by 41% of transmen and 2% of transwomen.

**Conclusions:** Prevalence of GCT differed across subgroups of participants and was lower than corresponding estimates reported elsewhere. The variability of results may reflect differences in recruitment procedures and response rates; however, it is also possible that it may be driven by geographic, socioeconomic, and health-related heterogeneity of the transgender population.

## Introduction

Medical gender confirmation therapy (GCT) includes three main types of interventions: treatment with hormones of the desired gender, surgical interventions to change the appearance of the chest or other secondary sex characteristics, and genital sex reassignment surgery.^1^ Despite reported importance of GCT,^2–4^ access to qualified healthcare professionals who know how to administer hormonal therapy (HT) or perform appropriate surgical procedures remains a major problem in many countries, including the United States.^5,6^

A number of studies evaluated GCT receipt among patients of specialized transgender care clinics.^7–11^ In addition, several recent surveys conducted in nonclinical settings found that the proportion of participants who reported receiving GCT ranged from 55% to 93%.^5,12–15^ Besides estimating GCT prevalence, some of those studies also tested the association of GCT with psychosocial health problems, smoking, and substance use. By contrast, data regarding determinants of GCT receipt or desire to undergo medical gender confirmation are relatively scant.^16,17^

In the present study, we sought to assess factors associated with previously received, current, or planned GCT in a sample of individuals who visited social media sites of a major transgender networking organization. The goal of the analysis was to examine GCT prevalence both overall and across various categories defined by gender identity and other demographic or personal characteristics.

## Methods

Data for the study were obtained from a one-time cross-sectional online survey about transgender health conducted by Emory University. The survey was approved by institutional review board and was administered among persons who visited the social media sites of the Southern Comfort Conference (SCC), a transgender education and social networking organization. A link to the survey was posted on the SCC website or Facebook page from October 2012 through the end of 2013. The survey was entirely anonymous. Once participants accessed the study website, they were taken through an informed consent process to ensure their understanding of the study purpose, procedures, risks and discomforts, benefits, and confidentiality.

Individuals were considered eligible for the survey if they indicated that they were at least 18 years of age and that their gender identity was different from the sex category assigned at birth. Persons whose birth sex was the same as current gender identity, those who reported being born with intersex conditions, and those who self-identified as gender nonconforming, but not explicitly transmen or transwomen, were excluded from the current analysis (*n*=89). All of these individuals skipped GCT-related questions.

After excluding individuals who skipped treatment-related questions, 280 of 453 eligible participants (62%) provided data for the final analysis. Persons who responded to treatment questions and those who did not report GCT information were of similar age, but respondents were more likely to be college educated (67% vs. 43%) and included a lower proportion of transmen (39% vs. 61%).

Respondents were asked about their sex assigned at birth and about their current gender identity. Additional questions collected information about participants' race, education, health insurance, and relationship status. The survey also asked the participants to identify various GCT types (HT, chest surgery, and genital reconstruction) that they previously received or were planning to receive. In addition to the structured survey questions, study participants were offered free text fields to share their thoughts about barriers that may prevent them from receiving desired GCT.

The dependent variables in the current study were receipt or having plans to receive HT, genital, or chest surgery. The independent variables of interest were current gender identity, age, race, education, relationship status, and health insurance. With respect to race, participants were categorized as Whites versus Other. The “Other” group included persons who self-identified as American Indian or Alaska Native, Asian, Black, multiracial, or those who declined to be associated with any group. Only 11 persons self-identified as Hispanic/Latino (seven among Whites). For this reason, Hispanics/Latinos were not considered as a separate group in the analyses.

Persons who reported receiving a 4-year college or a graduate degree were compared to those who did not complete college. Relationship status was categorized as single, married/in civil union, or in other committed partnership. Health insurance status was dichotomized as none versus any.

Transmen and transwomen were compared with respect to frequency and distribution of various GCT types, as well as demographic and personal characteristics. Multivariable analyses for each GCT type were carried out using logistic regression models that included gender identity, race, age (dichotomized at the median of 50 years), health insurance, relationship status (dichotomized as single vs. not single), and college education. Age and relationship status were included in the models as binary variables to allow sufficient sample sizes of the strata. The corresponding analyses for planned GCT included the same independent variables and covariates, but were restricted to persons who did not report ever receiving the treatment of interest. Each model was examined for two-way interactions between gender identity and each of the covariates to assess whether the associations with GCT differed in transmen and transwomen and needed to be reported separately.^18^ The results of multivariable analyses were expressed as adjusted odds ratios (AORs) and corresponding 95% confidence intervals (CIs). All analyses were performed using IBM SPSS Statistics for Windows, Version 20.0. (Armonk, NY; IBM Corp., 2011).

## Results

Of 280 study participants, 84% (*n*=234) were transwomen and 16% (*n*=46) were transmen (Table 1). Transmen had a greater proportion of persons under the age of 40 years (54%) compared to transwomen (20%). Over 90% of transwomen self-identified as Whites compared to 61% of transmen. A greater proportion of transwomen than transmen (50% vs. 24%) reported being married or in a civil union.

**Table 1.** **Demographic and Personal Characteristics of Study Participants**

	Total (*N*=280)		Transwomen (*N*=234)		Transmen (*N*=46)		
Variables^a^	*N*	%	*N*	%	*N*	%	*p*^b^
Age							
<40 years	71	25	46	20	25	54	<0.01
40–49 years	70	25	57	24	13	28	
50+ years	139	50	131	56	8	17	
Race							
White	241	86.1	213	91	28	61	<0.01
Other/mixed/undeclared^c^	39	13.9	21	9	18	39	
College education							
No	117	41.8	102	44	15	33	0.17
Yes	163	58.2	132	56	31	67	
Health Insurance							
No	37	13	30	13	7	15	0.66
Yes^d^	243	87	204	87	39	85	
Relationship status							
Single	111	40	95	41	16	35	<0.01
Married/in civil union	129	46	118	50	11	24	
In other committed partnership	40	14	21	9	19	41	

^a^ All categories are mutually exclusive.

^b^ Based on chi-square tests.

^c^ Includes persons who self-identified as American Indian or Alaska Native (*N*=3), Asian (*N*=5), Black (*N*=13), multiracial (*N*=13), or those who declined to be associated with any group (*N*=5).

^d^ Includes health management organization or private insurance with or without Medicare (*N*=186), Veterans Affairs or other military coverage (*N*=16), insurance from Canada (*N*=6), Medicare/Medicaid only (*N*=22), or any combination of at least two types of health insurance (*n*=13).

As shown in Table 2, ever and current use of HT was reported in 59% and 52% of all participants, respectively. The corresponding percentages of ever and current HT use were 58% and 47% for transwomen and 63% and 57% for transmen. Among 116 participants who never had HT, plans to receive hormones were reported in 52 (53%) of 99 transwomen and 13 (76%) of 17 transmen. More than a quarter of transmen reported previous chest surgery compared to only 5% of transwomen. When the analyses were restricted to persons with no history of previous chest surgery (*N*=257), plans to undergo the procedure were reported in 30 of 34 (88%) transmen and 89 of 223 (40%) transwomen.

**Table 2.** **Healthcare and Treatment-Related Characteristics of Study Participants**

	Total (*N*=280)		Transwomen (*N*=234)		Transmen (*N*=46)		
Variables	*N*	%	*N*	%	*N*	%	*p*^a^
Hormonal treatment							
Ever use	164	59	135	58	29	63	0.50
Current use	145	52	109	47	26	57	0.22
Planning to use^b^	65	56	52	53	13	76	0.07
Chest surgery							
Underwent	23	8	11	5	12	26	<0.01
Planning to undergo^c^	119	46	89	40	30	88	<0.01
Genital surgery							
Underwent	11	4	11	5	0	0	0.22^d^
Planning to undergo^e^	115	43	101	45	14	30	0.06

^a^ All *p* values are based on chi-square tests, unless otherwise indicated.

^b^ Analyses are limited to 116 persons (99 transwomen and 17 transmen) who never had HT.

^c^ Analyses are limited to 257 persons (223 transwomen and 34 transmen) who never had chest surgery.

^d^ Based on Fisher's exact test.

^e^ Analyses are limited to 269 persons (223 transwomen and 46 transmen) who never had genital surgery.

HT, hormonal therapy.

Only 11 persons, all transwomen, had genital surgery. Among study survey respondents who never had genital surgery, the proportions of transwomen and transmen planning to have this type of GCT were 45% (101 of 223) and 30% (14 of 46), respectively (*p*=0.06).

None of the interaction terms in the logistic regression models was statistically significant and for this reason only no-interaction models were used. Results of multivariable analyses that used HT and chest surgery receipt as the outcomes of interest are presented in Table 3. The equivalent analyses for genital surgery could not be conducted because none of the transmen in this study reported undergoing the procedure. Compared to white participants, those of “other, mixed, or undeclared” race were more likely to have ever received or were currently receiving HT. Having college education was associated with increased likelihood of HT, but the result was statistically significant only for current treatment (AOR=1.69, 95% CI: 1.02–2.80). Compared to participants who were single, those in a committed relationship of any kind were less likely to report ever receiving HT. Compared to participants under the age of 50, those who were 50 years of age or older were more than 3 times as likely to have had chest surgery (AOR=3.48, 95% CI: 1.09–11.14). Compared with transwomen, transmen were more than 12 times as likely to have had chest surgery (AOR=12.57, 95% CI: 3.95–40.01).

**Table 3.** **Multivariable Analyses of Factors Associated with Receipt of GCT Among All Study Participants (*N*=280)**

	Ever HT			Current HT			Underwent chest surgery		
Variables	OR^a^	95% CI^a^	*p*^a^	OR	95% CI^a^	*p*^a^	OR^a^	95% CI^a^	*p*^a^
Age									
<50 years	1	(Reference)		1	(Reference)		1	(Reference)	
50+ years	1.05	0.63–1.78	0.84	1.04	0.62–1.75	0.87	**3.48**	**1.09–11.14**	**0.04**
Race									
White	1	(Reference)		1	(Reference)		1	(Reference)	
Other/mixed/undeclared^b^	**2.75**	**1.19–6.38**	**0.02**	**2.38**	**1.11–5.13**	**0.03**	0.81	0.21–3.19	0.77
Education									
No college degree	1	(Reference)		1	(Reference)		1	(Reference)	
College degree or more	1.34	0.81–2.23	0.26	**1.69**	**1.02–2.80**	**0.04**	2.80	0.86–9.12	0.09
Relationship status									
Single	1	(Reference)		1	(Reference)		1	(Reference)	
Not single^c^	**0.59**	**0.36–0.99**	**0.05**	0.65	0.39–1.07	0.09	1.81	0.62–5.27	0.28
Insurance status									
No insurance	1	(Reference)		1	(Reference)		1	(Reference)	
Any insurance^d^	1.26	0.60–2.67	0.55	1.06	0.50–2.22	0.88	0.57	0.14–2.44	0.45
Gender identity									
Transwomen	1	(Reference)		1	(Reference)		1	(Reference)	
Transmen	0.97	0.46–2.02	0.93	1.15	0.56–2.36	0.70	**12.57**	**3.95–40.01**	**<0.01**

^a^ Bold print indicates statistically significant results at two-sided alpha level of 0.05.

^b^ Includes persons who self-identified as American Indian or Alaska Native (*N*=3), Asian (*N*=5), Black (*N*=13), multiracial (*N*=13), or those who declined to be associated with any group (*N*=5).

^c^ Includes persons who are married, in civil union, or other committed relationship.

^d^ Includes health management organization or private insurance with or without Medicare (*N*=186), Veterans Affairs or other military coverage (*N*=16), insurance from Canada (*N*=6), Medicare/Medicaid only (*N*=22), or any combination of at least two types of health insurance (*n*=13).

CI, confidence interval; GCT, gender confirmation therapy; OR, odds ratio.

Table 4 summarizes results for each planned GCT type among persons who never had this treatment. Participants who were at least 50 years of age were less likely to plan GCT than younger survey respondents, with significant results for HT (AOR=0.25; 95% CI: 010–0.63) and genital surgery (AOR=0.36; 95% CI: 0.21–0.62), but not for chest surgery (OR=0.65; 95% CI: 0.37–1.14). Using single participants as the reference group, those who reported being in a committed relationship of any kind were significantly less likely to plan GCT, and the association was consistent across all three treatment types. Plans for HT did not differ in transmen and transwomen, but gender identity was associated with plans for surgical treatment. Compared to transwomen, transmen were more likely to plan chest surgery (AOR=11.45; 95% CI: 3.62–36.20), but were less likely to plan genital surgery (AOR=0.30; 95% CI: 0.14–0.68).

**Table 4.** **Multivariable Analyses of Factors Associated with Planned GCT Type Among Study Participants Who Never Had That Treatment**

	Planned HT^a^			Planned chest surgery^a^			Planned genital surgery^a^		
	*N*=116			*N*=257			*N*=269		
Variables	OR^b^	95% CI^b^	*p*^b^	OR^b^	95% CI^b^	*p*^b^	OR^b^	95% CI^b^	*p*^b^
Age									
<50 years	1	(Reference)		1	(Reference)		1	(Reference)	
50+ years	**0.25**	**0.10–0.63**	**<0.01**	0.65	0.37–1.14	0.13	**0.36**	**0.21–0.62**	**<0.01**
Race									
White	1	(Reference)		1	(Reference)		1	(Reference)	
Other/mixed/undeclared^c^	0.69	0.12–3.88	0.67	0.78	0.32–1.90	0.58	1.67	0.75–3.69	0.21
Education									
No college degree	1	(Reference)		1	(Reference)		1	(Reference)	
College degree or more	2.03	0.82–5.03	0.13	0.87	0.51–1.51	0.63	0.76	0.45–1.28	0.30
Relationship status									
Single	1	(Reference)		1	(Reference)		1	(Reference)	
Not single^d^	**0.38**	**0.15–0.99**	**0.05**	**0.47**	**0.27–0.82**	**<0.01**	**0.56**	**0.33–0.96**	**0.03**
Insurance status									
No insurance	1	(Reference)		1	(Reference)		1	(Reference)	
Any insurance^e^	0.13	0.02–1.10	0.06	0.62	0.27–1.39	0.25	0.75	0.34–1.66	0.48
Gender identity									
Transwomen	1	(Reference)		1	(Reference)		1	(Reference)	
Transmen	1.87	0.49–7.10	0.36	**11.45**	**3.62–36.20**	**<0.01**	**0.30**	**0.14–0.68**	**<0.01**

^a^ Limited to participants who never had that treatment.

^b^ Bold print indicates statistically significant results at two-sided alpha level of 0.05.

^c^ Includes persons who self-identified as American Indian or Alaska Native, Asian, Black, multiracial, or those who declined to be associated with any group; numbers differ depending on the analysis because analysis for each planned treatment is limited to participants who never had that treatment.

^d^ Includes persons who are married, in civil union, or other committed relationship.

^e^ Includes health management organization or private insurance with or without Medicare, Veterans Affairs or other military coverage, insurance from Canada, Medicare/Medicaid only, or any combination of at least two types of health insurance; numbers differ depending on analysis because analysis for each planned treatment is limited to participants who never had that treatment.

**FIG. 1.**
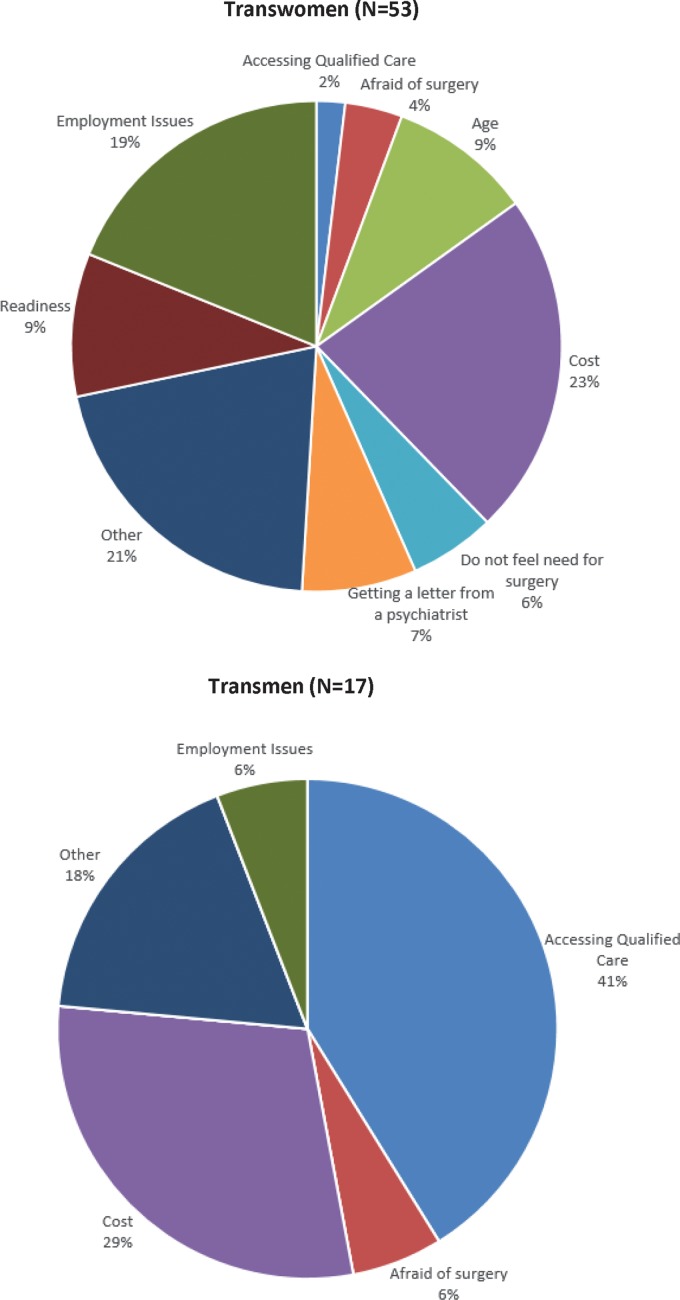
Barriers to receiving GCT reported by study participants. GCT, gender confirmation therapy.

Only a subset of survey participants used the free text option to report on barriers to receiving GCT (*N*=17 for transmen and *N*=53 for transwomen). Figure 1 compares the relative frequencies of GCT barriers reported by transmen and transwomen. Treatment cost was named as the main problem by 23% of transwomen and 29% of transmen. Accessing a qualified healthcare provider for transgender-related care was listed as the primary reason for not receiving surgery by 41% of transmen and 2% of transwomen. Receiving a letter from a psychiatrist as approval to receive the surgery was listed as a primary reason for not receiving surgery by 8% of transwomen participants and none of the transmen.

## Discussion

Approximately, one-half (52%) of our study participants reported currently receiving HT and less than 10% reported receiving any type of GCT surgery. These percentages are lower than those reported in previous studies.^5,7–15,19–24^ Previous studies assessing prevalence of GCT receipt fall into two categories: those conducted in clinics and those that recruited participants in nonclinical settings.

Studies conducted in clinics would, *ipso facto*, overestimate GCT rates in the general community of transgender people. For example, in a study conducted in Ghent, Belgium, nearly all participants were receiving hormonal treatment and nearly three-quarters (73%) underwent genital surgery; however, that study enrolled only persons who received the diagnosis of gender dysphoria and limited inclusion to only those patients who ever received HT for at least 3 months.^21^ A more recent publication from Ireland was also limited to gender dysphoria patients, who were receiving or were referred for HT.^19^ In another clinic-based study that recruited participants from psychiatric departments at teaching hospitals in France, the estimated prevalence of HT was 73%.^8^

A few studies examined prevalence of GCT in primary care clinics. One such study of transwomen in New York City reported that 70% of participants used hormones at the time of the interview.^20^ A 1999 study conducted in Scotland reported prevalence estimates for HT use that were similar to those observed in our survey; however, data for that study were collected from a questionnaire administered among physicians in general medical practices.^22^ It is not entirely clear if general practitioners surveyed in that study were able to fully ascertain GCT treatment receipt among their transgender patients.

A number of studies estimated prevalence of GCT in nonclinical samples of transgender people in the United States. Nuttbrock et al. examined 230 transwomen identified through support organizations, the internet, newspaper, advertisements, and other nonclinical sources in the New York metropolitan area. Although the analysis in that study focused on mental health issues, the authors also reported that the prevalence of HT use in the previous 6 months in their study group was 52%, an estimate that is very similar to ours.^24^

Other studies based on nonclinical samples reported higher prevalence estimates. The largest survey in this category was administered through direct contacts with transgender-led or transgender-serving community organizations in the United States.^5^ Among ∼6500 survey participants (both transmen and transwomen), 61% reported having “medically transitioned” and 33% indicated that they had “surgically transitioned.” A separate analysis of the same data, but limited to transwomen, reported prevalence estimates of 63% for HT and 31% for surgery.^14^

Two studies utilizing nonclinical sampling methods were conducted in the San Francisco Bay Area. The Transfemales Empowered to Address Community Health (TEACH) study recruited participants using respondent-driven sampling. Of the 314 transwomen in the sample, 93% had accessed hormones, 15% had breast augmentation, and 9% had genital surgery.^15^ These estimates are almost twice as high as those observed in our data. A more recent study conducted using venue-based recruitment and flyer postings involved a one-time survey of 241 transwomen.^12^ Current hormone use in that study was reported in 70% of participants, which is also higher than the corresponding estimate of 47% in our study.

An important feature of this study is the focus on the prevalence of different GCT types across subgroups of transgender people. The gender identity difference in genital surgery receipt was not statistically significant in this study population; however, it is worth pointing out that all persons who reported having this type of surgery were transwomen and that plans to undergo this type of surgery were far more common in transwomen than transmen. These differences are likely attributable to the greater technical difficulties and higher rate of complications associated with that of female-to-male relative to male-to-female genital surgery.^25,26^ It is notable that transwomen in our study were far more likely than transmen to identify inadequate access to qualified healthcare providers as the primary reason for not receiving surgery. As insurance coverage and the number of surgeons trained to perform the procedure continue to increase, it is expected that more transmen will be undergoing genital gender confirmation.

Transmen in our study were 12 times more likely to report having had chest surgery relative to transwomen. It is important to keep in mind that chest surgery is considered an essential step toward improving body image in transmen.^27,28^ By contrast, in transwomen, a certain degree of breast augmentation can be achieved by HT alone.^29^

Having health insurance status was not associated with GCT in this study. This finding is in keeping with the fact that many insurance companies in the United States did not cover GCT at the time of this survey.^29^ Both current HT and chest surgery were more frequently reported among persons with at least a college degree. It is likely that education in this population served as a surrogate measure of income, which was not directly measured in our study. Cost was one of the main barriers to receiving desired treatment in our study and also reported as an important obstacle to GCT in previous studies.^5^

These observations indicate that determinants of GCT receipt are complex, multifactorial, and likely differ across population subgroups. For this reason, future studies should take into consideration additional patient-, provider-, and system-related barriers to and facilitators of access to GCT. More detailed data are needed on participants' level of gender dysphoria, level of income, social support, and history of receiving primary and specialized care.

Perhaps the most important limitation of the present study is a relatively large proportion (38%) of subjects who declined to respond to the treatment questions. Among eligible transgender persons, those who responded to treatment questions and those who did not report GCT information differed with respect to college education (67% vs. 43%) and the proportion of transmen (39% vs. 61%). This differential reporting warrants caution, particularly when interpreting some of the weaker associations. Exclusions of 89 noneligible persons who reported being born with intersex conditions, and those who self-identified as gender nonconforming, did not affect the results because all of these individuals skipped GCT-related questions.

Whenever an online survey is conducted, there is a concern about a need for identification and deduplication of redundant or fraudulent responses.^30^ This concern is particularly justified when respondents are offered incentives for participation. As our survey did not offer any incentives, the likelihood of someone taking it multiple times is reduced.

As several study subgroups included relatively few participants, our analyses lacked detailed categorization for some of the variables. For instance, due to small numbers of participants representing racial and ethnic minorities, several race categories had to be combined into a single group. Similarly, sparse stratum-specific data for insurance and relationship status required that they be analyzed as binary variables.

The impact of the above limitations on the study results is difficult to ascertain, but it is clear that some of the observed associations require independent confirmation before definitive conclusions are drawn. Very strong and statistically significant results such as the association between gender identity and chest surgery are unlikely to be explained by nonresponse or analysis choices and most likely represent a true phenomenon, although the magnitude of the observed association may have been affected by various sources of bias.

It is likely that our participants who were recruited from a single social network were different from the general population of transgender people. Although the social network used for the study recruitment was not limited to persons living in the southern United States, it is possible that these findings are more reflective of regional patterns. The relatively high level of education and the predominance of Non-Hispanic Whites and transwomen raise additional concerns about the generalizability of our findings. The above considerations notwithstanding, the present study adds to the literature on transgender people identified in nonclinical settings.

In summary, past receipt and plans to receive various surgical GCT types differ by gender identity. The gender identity difference is far less pronounced for HT. The data also suggest that socioeconomic status (as measured by level of education) may act as a determinant of GCT receipt. As coverage for HT and surgery continues to increase, it would be important to reexamine the association between GCT and health insurance status. Other studies designed to achieve better rates of questionnaire completion, higher statistical power, and greater geographical representation are needed to more fully understand barriers and facilitators of adequate gender confirmation care. The disagreement between our results and those reported in other surveys may reflect differences in recruitment procedures and response rates; however, it is also possible that it may be driven by geographic, socioeconomic, and health-related heterogeneity of the transgender population.

## Abbreviations Used

AOR: adjusted odds ratio
CI: confidence interval
GCT: gender confirmation therapy
HT: hormonal therapy
SCC: Southern Comfort Conference
